# Characterization of Oligodendroglial Populations in Mouse Demyelinating Disease Using Flow Cytometry: Clues for MS Pathogenesis

**DOI:** 10.1371/journal.pone.0107649

**Published:** 2014-09-23

**Authors:** Andrew P. Robinson, Jane M. Rodgers, Gwendolyn E. Goings, Stephen D. Miller

**Affiliations:** 1 Department of Microbiology-Immunology, Feinberg School of Medicine, Northwestern University, Chicago, IL, United States of America; 2 Interdepartmental Immunobiology Center, Feinberg School of Medicine, Northwestern University, Chicago, IL, United States of America; University of Muenster, Germany

## Abstract

Characterizing and enumerating cells of the oligodendrocyte lineage (OLCs) is crucial for understanding demyelination and therapeutic benefit in models of demyelinating disease in the central nervous system. Here we describe a novel method for the rapid, unbiased analysis of mouse OLCs using flow cytometry. The assay was optimized to maximize viable yield of OLCs and maintain OLC antigen integrity. Panels of antibodies were assembled for simultaneous analysis of seven antigens on individual cells allowing for characterization of oligodendroglial cells throughout the lineage. We verified the utility of the assay with cultured OLCs and through a time course of developmental myelination. Next we employed the assay to characterize OLC populations in two well-characterized models of demyelination: cuprizone-induced demyelination and experimental autoimmune encephalomyelitis (EAE). In EAE we observed a dramatic loss of mature oligodendrocytes coincident with a dramatic expansion of oligodendrocyte progenitors cells (OPCs) at the onset of disease suggesting an attempt of the host to repair myelin. This expanded OPC pool was maintained through remission and relapse suggesting an arrest in differentiation in the face of the chronic autoimmune T cell-mediated inflammatory response. These robust, reproducible changes in OLCs through disease provide a rapid quantitative global analysis of myelin-producing cells in the adult mouse brain and important information regarding effects of disease on oligodendroglial proliferation/differentiation which is useful for defining the pathogenesis and therapy of MS.

## Introduction

Oligodendrocytes in the central nervous system (CNS) produce a complex lipid and lipoprotein-rich insulating sheath termed myelin that supports electrical conduction in neurons [1]. Genetic abnormalities in developmental myelination can be lethal, and adult demyelination or loss of the myelin sheaths can produce severe clinical disabilities. Multiple sclerosis (MS), the hallmark demyelinating neurodegenerative disease, is an autoimmune-mediated disorder characterized by multifocal inflammatory lesions of demyelination [2], [3]. The resulting oligodendrocyte destruction and axonal impairment can produce debilitating motor, sensory, and cognitive deficits. The brain has a robust capacity to regenerate damaged myelin, that is resident oligodendrocyte progenitor cells (OPCs) mature and form new myelin in a dynamic process known as remyelination. In MS patients the remyelination process ultimately fails to fully correct for myelin loss and resulting clinical deficits even in the absence of inflammation and immune cell infiltration [4]. There are a number of experimental rodent models of demyelinating disease including cuprizone-induced demyelination and experimental autoimmune encephalomyelitis (EAE), and remyelination has been described in both of these models [5], [6].

Characterizing de- and remyelination in EAE has proven surprisingly difficult as the spatial and temporal variability between demyelinating lesions is high. Lesions are interspersed throughout the CNS and do not necessarily occur in the same anatomical regions [7]. Within the lesion myelin loss and new myelin formation occur in a dynamic process and can vary significantly between lesions [8], [9]. Inter-animal variability adds another level of complexity as clinical deficits and disease pathology can vary in timing and severity among a cohort.

At present de- and remyelination are almost wholly characterized by histology, and remyelination is only definitively distinguished at the electron microscopic (EM) level. These assays routinely use oligodendrocyte antibodies that label antigens on myelin processes as well as cell bodies making the ability to distinguish individual cells from background staining, and thus quantification, difficult. For animal models of demyelination such as EAE these assays are time-consuming and prone to high statistical variance and subjectivity given the spatial, temporal, and inter-animal variability of the disease. Many lesions must be identified, characterized throughout, and in multiple animals to begin to approach statistical significance. Dynamic, statistically relevant analysis of de- and remyelination throughout a disease course using histological techniques approaches unwieldy levels of time and resources.

We investigated whether cells throughout the oligodendroglial lineage (OLCs) can be reliably and rapidly quantified during de- and remyelination in the mouse CNS by flow cytometry. This technique allows for analyzing protein expression on the cellular level by suspending cells from dissociated tissue in a buffered solution and analyzing single cells with an optical detection apparatus [10]. Individual cells are excited by laser light and spectral emission is detected using specific filters and a series of photomultiplier tubes (PMTs). Inherent spectral characteristics as well as proteins labeled with fluorescent dyes or antibodies can be detected and analyzed. There is a broad range of commercially available antibodies, fluorochromes, and detection filter configurations allowing for routine analysis of ≥ eight proteins expressed by a single cell. Rapid acquisition (thousands of cells per second) allows for analysis of ≥1×10^6^ cells to produce a global profile of cell populations within a tissue. Routinely used to analyze circulating hematopoietic cells from patients and animal models of human disease, the technique remains largely unexplored for characterization of resident CNS cells [10].

## Methods

### Mice

Female C57BL/6 and SJL/J mice were purchased from Harlan Laboratories (Bethesda, MD). The mice were housed under specific pathogen-free conditions in the Northwestern University Center for Comparative Medicine barrier facility (Chicago, IL). All protocols (2010-0357) were approved by the Northwestern University Institutional Animal Care and Use Committee in accordance with Federal Animal Welfare Regulations.

### Antibodies

All antibodies, fluorochromes, and staining concentrations are detailed in **Table 1**. For many experiments purified antibodies were conjugated to fluorochromes using Lightning-Link Antibody Labeling Kits (Novus Biologicals, Littleton, CO) as per manufacturer's instructions. Briefly fluorochromes were covalently linked to primary antibodies in a proprietary binding solution near neutral pH then unconjugated fluorochromes were quenched. Fluorochromes used include APC, APC-Cy7, Atto488, Atto700, FITC, PerCP-Cy5.5, PE-Cy7, and R-PE.

**Table 1:** Antibodies utilized for oligodendrocyte lineage cell characterization by flow cytometry.

Antigen	Ig Species and Class	Clone	Fluorochrome conjugated	Staining concentration	Manufacturer	RRID
A2B5	Mouse IgM	105	PE-Cy7	0.5 µg/test	R&D Systems	AB_357687
CD45	Rat IgG2b	30-F11	V450 or V500	0.2 µg/test	BD Biosciences	AB_10697046
GALC	Mouse IgG3	Unspecified monoclonal	PE	1 µg/test	Millipore	AB_94857
MOG	Mouse IgG1	8-18C5	APC-Cy7 or Atto700	1 µg/test	Millipore	AB_1587278
MOSP	Mouse IgM	Unspecified monoclonal	PerCP	1 µl/test (concentration unspecified)	Millipore	AB_2145545
NG2	Mouse IgM	Unspecified monoclonal	PerCP-Cy5.5	1 µg/test	Millipore	AB_91789
O1	Mouse IgM	O1	APC-Cy7	0.5 µg/test	R&D Systems	AB_357618
O4	Mouse IgM	81	APC	1 µg/test	Millipore	AB_94872
PDGFRα	Rat IgG2a	APA5	FITC or Atto488	2.5 µg/test	Millipore	AB_2283679

### Optimizing enzymatic dissociation

C57BL/6 mice were anesthetized with 50 mg/kg pentobarbital and transcardially perfused with 30 ml PBS. Brains and spinal cords were dissected and coarsely chopped with microdissecting scissors. 1 ml of one of nine dissociation enzymes (**Table S1**) was added to individual brain and spinal cord tissues and incubated for 30 min at 37°C. 2 ml 10% FBS in Hank's Balanced Salt Solution (HBSS) was added to each sample, and tissue was manually triturated with a transfer pipette. Samples were transferred to 100 µm cell strainers (BD Biosciences, San Jose, CA) and pushed through using syringe plungers. The cell strainers were thoroughly rinsed with ∼15 ml 10% FBS in HBSS and cell suspensions were centrifuged. To purify cells from myelin debris, cells were resuspended in 40% Percoll (Amersham, Piscataway, NJ) in HBSS and centrifuged at 650×g without brake for 25 min at room temperature. The myelin top layer was aspirated and mononuclear cells were resuspended in flow cytometry buffer (1% normal mouse serum, 1% normal rat serum, 2 mM EDTA in DPBS (Dulbecco's phosphate buffered saline)). Cells were counted using a Coulter Counter (Beckman Coulter, Brea, CA) and 10^6^ cells per sample were stained for analysis. Excess cell suspensions were combined for unstained and single stained controls. Live cells were stained with Calcein Violet AM and dead cells with an amine-reactive aqua-fluorescent dye (Life Technologies) in Ca^++^ and Mg^++^-free PBS for 10 min on ice. Cells were washed twice and resuspended in flow cytometry buffer. Samples were run on a FACSCanto flow cytometer (BD Biosciences) and analyzed using FlowJo software (Tree Star, Ashland, OR, RRID:nif-0000-30575). Results are plotted as percent of total cells.

To assay antigen integrity following enzymatic dissociation, brains and spinal cords were dissected, incubated in dissociation enzymes, and single cells isolated from myelin debris as before. Cells were counted, and 10^6^ cells per sample were stained for analysis. Fc receptors were blocked with anti-mouse CD16/32 (0.25 µg; eBioscience, San Diego, CA, Cat# 14-0161-86 RRID:AB_467135) for 15 min on ice. Live cells were labeled with Calcein Violet AM. Cells were then stained with anti-A2B5, O4, O1, or respective isotype in flow cytometry buffer for 30 min at 4°C. All antibodies were conjugated to FITC beforehand as previously described. Cells were washed twice and resuspended in flow cytometry buffer. Samples were run on a FACSCanto flow cytometer and analyzed using FlowJo software. Results are plotted as percent of total live cells or absolute cell number as backcalculated using total cell counts.

### Optimizing cell purification

Brains and spinal cords were dissected and chopped as before. 1 ml Accutase was added to brain and spinal cord tissues and incubated for 30 min at 37°C. 2 ml 10% FBS in HBSS was added to each sample, tissue was triturated and filtered, and samples were centrifuged as before. For cell purification suspensions were resuspended in 30% Percoll and layered over 70% Percoll or resuspended in 40% Percoll in HBSS and centrifuged at 650×g without brake for 25 min at room temperature. In both methods the myelin top layer was transferred to a new tube. For the 30/70% gradient cells were isolated from the interface and pellet. For the 40% Percoll cells were isolated from the pellet. Alternatively single cells were purified using Myelin Removal Beads (Miltenyi Biotec, Auburn, CA) according to the manufacturer's instructions. Following all purification methods, samples were resuspended in flow cytometry buffer. Cells were counted, and 10^6^ cells per sample were stained for analysis. Excess cell suspensions were combined for control staining. Fc receptors were blocked, and live cells were labeled with Calcein Violet AM as before. Cells were then stained with anti-A2B5, PDGFRα, NG2, O4, GALC, MOG, or respective isotype antibodies in flow cytometry buffer for 30 min at 4°C. All antibodies were previously conjugated to FITC. Cells were washed twice and resuspended in flow cytometry buffer. Samples were run on a FACSCanto flow cytometer and analyzed using FlowJo software. Results are plotted as absolute cell number as backcalculated using total cell counts.

### Primary mouse OPC isolation, culture, and flow cytometry

OPCs were isolated and cultured from mice according to previously described immunopanning protocols from B. Barres with minor modifications [11], [12]. Briefly, brains were extracted from five to seven day old C57BL/6 pups and dissociated in 200 U buffered papain (Worthington Biochemical, Lakewood, NJ) for 30 minutes at 37°C in 95% O_2_, 5% CO_2_. Papain was quenched with 1.5 mg ovomucoid (Worthington Biochemical), the tissue was triturated into a single cell suspension, and the suspension was filtered through 100 µm cell strainers. OPCs were purified by sequential immunopanning: one negative selection plate for RAN2 followed by two positive selection plates for O4. Hybridoma supernatants used for coating immunopanning plates were a gift from B. Popko, University of Chicago. Cells were washed with DPBS, lifted with 0.25% trypsin from the positive selection plates, and plated at a density of 10^6^ cells per plate on poly-D-lysine (Sigma-Aldrich, St. Louis, MO) coated 10 cm^2^ plates in OPC growth medium with PDGF and culture conditions as previously described [12]. For oligodendrocyte differentiation, cultures were changed to differentiation medium without PDGF. Cultures were grown for six days before flow cytometric analysis. Purity of cultures was >98% as verified by the lack of GLAST and CD45 expression by flow cytometry.

For flow cytometric assays plates were washed with DPBS and cells lifted with 0.25% trypsin for 5 min. Trypsin was quenched with 30% FBS in DPBS, and cells were centrifuged and resuspended in flow cytometry buffer. Cells were counted, and 10^6^ cells per sample were stained for analysis. Excess cell suspensions were combined for control staining. Fc receptors were blocked as before. Cells were then stained with a cocktail of anti-A2B5, O4, O1, MOSP, and GALC in flow cytometry buffer for 30 min at 4°C. All antibodies were previously conjugated to unique fluorochromes. For control staining, single stains and a fluorescence minus one (FMO) strategy were used where all antibodies except one were added to individual control tubes. Cells were washed twice and resuspended in flow cytometry buffer. Samples were run on a FACSCanto flow cytometer and analyzed using FlowJo software. Results are plotted as percent of total live cells ± SD of individual cultures.

### qRT-PCR of sorted adult oligodendroglial populations

Brains from 40 adult naïve C57BL/6 mice were extracted, pooled, dissociated using Accutase, and purified using 40% Percoll as described above. Cells were counted, and an aliquot was removed for control single stains and FMO tubes. Fc receptors were blocked, and live cells were labeled with Calcein Violet AM as before. Cells were then stained with a cocktail of anti-A2B5, PDGFRα, NG2, O4, GALC, and MOG in flow cytometry buffer for 30 min at 4°C. Cells were washed twice and resuspended in flow cytometry buffer. Early OPCs (A2B5^+^PDGFRα^+^), intermediate OPCs (O4^+^NG2^+^), and mature oligodendrocytes (GALC^+^MOG^+^) were sorted by FACS into lysis/binding buffer (Miltenyi Biotec) using a FACSAria 5 (BD Biosciences). Sorted cells were stored at −80°C. After thawing on ice, total RNA was isolated using µMACS mRNA Isolation Kit (Miltenyi Biotec) and cDNA was synthesized on column with µMACS One-step cDNA Synthesis Kit (Miltenyi Biotec). Gene expression was analyzed by qRT-PCR using specific primers (Qiagen, Valencia, CA, **Table S2**), RT^2^ SYBR Green FAST Mastermix (Qiagen), and an iQ5 real-time PCR detection system (Biorad, Hercules, CA). Results were standardized to a housekeeping gene and normalized to gene expression in the A2B5^+^PDGFRα^+^ sorted early OPC population. Data is plotted as relative expression ± SD of technical replicates.

### Oligodendroglial analysis during mouse postnatal development

Oligodendroglial cells were isolated from two age-matched litters of C57BL/6 pups on postnatal days 4, 6, 7, 8, and 10 (n≥3/day). Brains were extracted, manually triturated, and single cell suspensions were filtered through 100 µm cell strainers. Cells were counted by haemocytometer and 10^6^ cells per sample were stained for analysis. Excess cell suspensions were combined for control single stain and FMO tubes. Fc receptors were blocked and live cells stained as before. Cells were then stained with a cocktail of anti-A2B5, NG2, GALC, and CD45 in flow cytometry buffer for 30 min at 4°C. Antibodies were previously conjugated to unique fluorochromes. Cells were washed twice and resuspended in flow cytometry buffer. Samples were run on a FACSCanto flow cytometer and analyzed using FlowJo software. Results are plotted as total cell number or percent of CD45^-/low^ CNS resident cells ± SD of individual mice.

### Oligodendroglial analysis during cuprizone-induced demyelination

Eight-week-old male C57BL/6 mice were fed 0.2% cuprizone (bis-cyclohexanone oxaldihydrazone, Sigma-Aldrich) in standard rodent chow. Mice were maintained on a cuprizone diet for five weeks then returned to normal chow for a sixth week. Once a week a cohort of mice (n = 5) and age-matched naïve control mice (n = 3) were exsanguinated. Brains were extracted, dissociated with Accutase, and purified using 40% Percoll. Cells were counted and 10^6^ cells per sample were stained for analysis. Excess cell suspensions were combined for control single stain and FMO tubes. Fc receptors were blocked and live cells stained as before. Cells were stained with a cocktail of anti-A2B5, PDGFRα, O4, O1, GALC, and CD45 in flow cytometry buffer for 30 min at 4°C. Antibodies were previously conjugated to unique fluorochromes. Cells were washed twice and resuspended in flow cytometry buffer. Samples were run on a FACSCanto flow cytometer and analyzed using FlowJo software. Absolute cell numbers were backcalculated using percent of CD45^-/low^ CNS resident cells and total cell counts. Data are plotted as mean ± SD of individual mice.

### Oligodendroglial analysis during relapsing-remitting EAE

Relapsing-remitting EAE was induced in 5-7-week-old SJL/J females as previously described [13]. Briefly mice were subcutaneously injected with an emulsion of complete Freund's adjuvant containing 200 µg *Mycobacterium tuberculosis* H37Ra (Difco, Detroit, MI) and 50 µg PLP_139–151_ peptide (Genemed Synthesis, San Antonio, TX) distributed in three spots on the flank. Mice were monitored daily for clinical symptoms of EAE and paralyzed animals were provided easier access to food and water. Mice were scored daily on a scale of 0–5 as follows: 0, no abnormality; 1, limp tail or hind limb weakness; 2, limp tail and hind limb weakness; 3, partial hind limb paralysis; 4, complete hind limb paralysis; and 5, moribund. At disease onset, peak, remission, and relapse a cohort of mice (n = 5) and age-matched naïve control mice (n = 3) were sacrificed. Spinal cords were extracted, dissociated with Accutase, and purified using 40% Percoll. Cells were counted and 10^6^ cells per sample were stained for analysis. Excess cell suspensions were combined for control single stain and FMO tubes. Fc receptors were blocked and dead cells stained as before. Cells were stained with a cocktail of anti-A2B5, PDGFRα, NG2, O4, GALC, MOG, and CD45 in flow cytometry buffer for 30 min at 4°C. Antibodies were previously conjugated to unique fluorochromes. Cells were washed twice and resuspended in flow cytometry buffer. Samples were run on a FACSCanto flow cytometer and analyzed using FlowJo software. Results are displayed as percent of CD45^-/low^ CNS resident cells ± SD of individual mice.

### Data analysis/statistics

All experiments were conducted a minimum of three times except cuprizone studies which were conducted twice. Prism 5 software (GraphPad, RRID:rid_000081) was used for all statistical analyses. For analysis of cultured cells, statistical comparisons were made using Student's two-tailed *t* test. For qRT-PCR studies sorted populations were compared using one-way ANOVA followed by Tukey's test for post-hoc analysis. For developmental myelination, cuprizone, and EAE studies cell populations in naïve and/or disease mice at different time points were compared to naïve control mice using one-way ANOVA followed by Tukey's test for post-hoc analysis.

## Results

**Figure 1:**
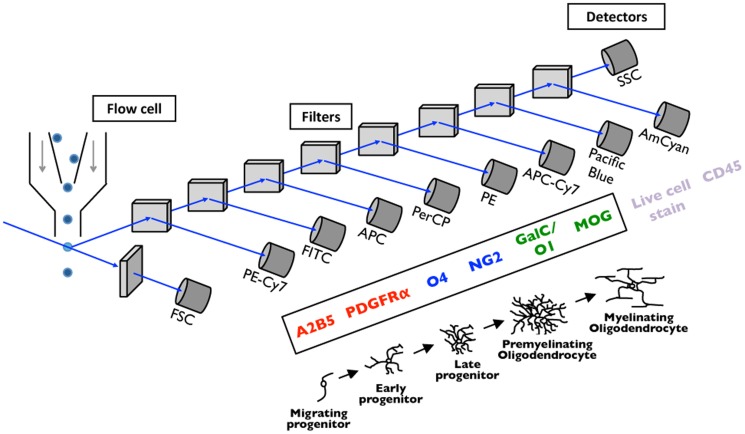
Flow cytometry for oligodendroglial analysis. Flow cytometry allows for rapid analysis of eight or more proteins expressed on individual cells. Cells are streamed through a narrow flow cell sequentially and interrogated individually, thus largely static organs such as the brain must be dissociated into individual cells prior to analysis. Cells are stained with a cocktail of antibodies, each conjugated to a unique fluorochrome. Because of distinct proteins changes that occur during the maturation of the oligodendrocyte, antibodies can be utilized to characterize cells throughout the oligodendrocyte lineage. Thousands of cells per second are streamed through the flow cytometer, excited by laser light, and emitted fluorescence is directed through filters to photomultiplier tubes specific for each fluorochrome. For subsequent analysis, single cells are distinguished from small debris using forward (FSC) and side (SSC) scatter of light. Live cells are distinguished from dead cells using a live/dead dye, and CNS resident cells are distinguished from residual hematopoietic cells using a CD45 antibody. The entire oligodendrocyte lineage, from early progenitor to mature oligodendrocyte, can be analyzed and quantified.

Our strategy for characterizing oligodendroglial populations in the mouse brain using flow cytometry is detailed in **Fig. 1**. CNS tissue is dissociated, and single cells are purified from cellular debris. Cells in suspension are stained with a cocktail of antibodies against proteins expressed through oligodendrocyte development. Each antibody was previously conjugated to a unique fluorochrome. Thousands of cells per second are flowed in a stream of buffer through the flow cytometer apparatus. Cells are individually interrogated by laser light, and emitted light is directed through a series of filters to photomultiplier tubes for specific fluorochromes. For analysis cells are identified by their forward and side scatter of light. To assay CNS resident cells, a live/dead cell stain is used to exclude dead cells and a CD45 antibody is used to exclude hematopoietic cells. By examining proteins expressed at distinct stages of oligodendrocyte development, the entire lineage from early oligodendrocyte progenitor cells to mature oligodendrocytes can be analyzed and enumerated.

### Optimization of CNS preparation for flow cytometric analysis

**Figure 2:**
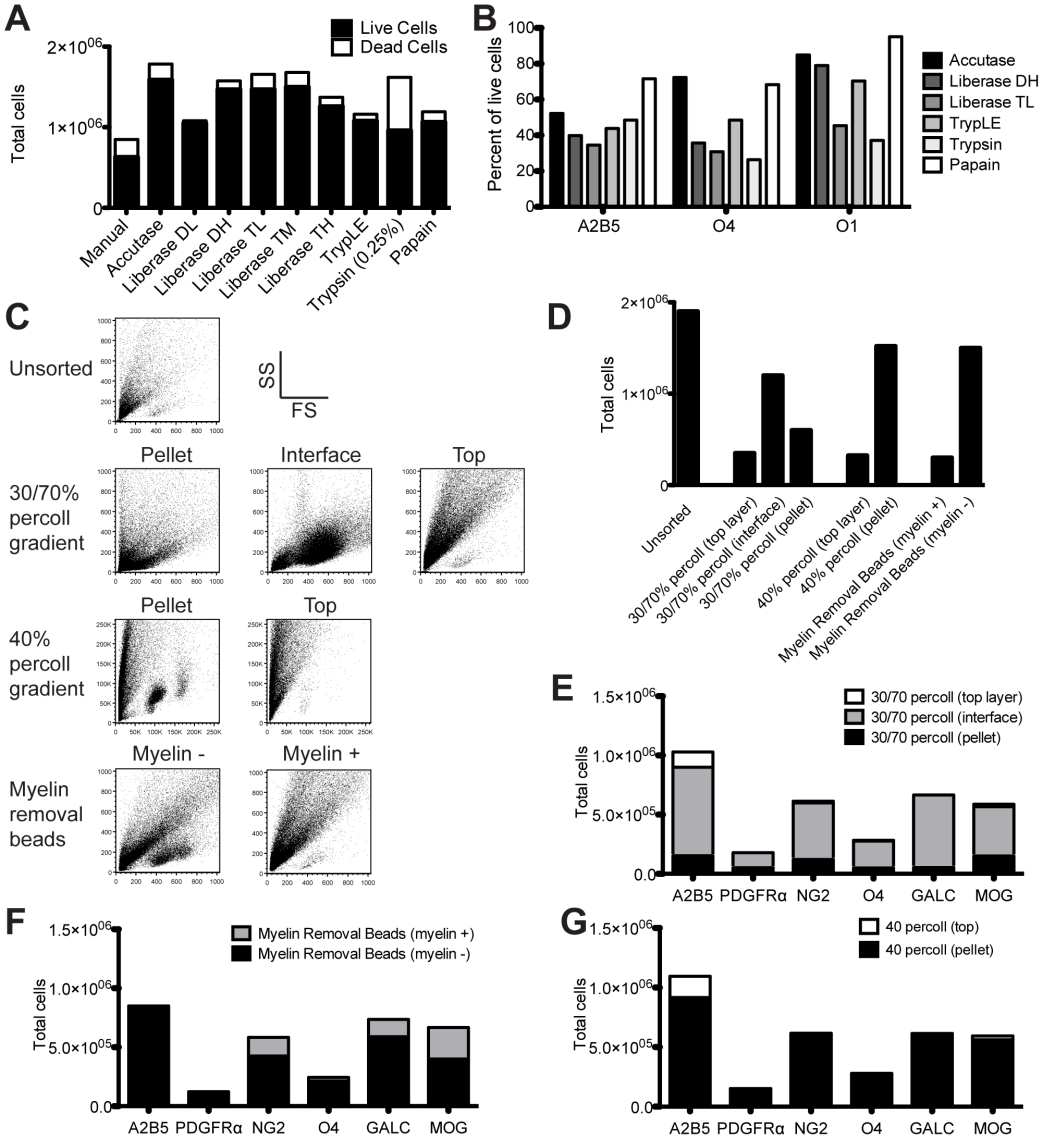
Optimization of oligodendroglial isolation and purification for flow cytometry. (**A**) Brains were removed from C57BL/6 mice, coarsely chopped, and dissociated with 1 ml of one of eight dissociation enzyme for 30 min at 37°C: Accutase, Liberase DL, Liberase DH, Liberase TL, Liberase TM, Liberase TH, TrypLE, 0.25% Trypsin-EDTA, or PBS. Digested tissues were filtered, and single cells were purified using density centrifugation. Total cells were calculated by haemocytometer. Live cells and dead cells were stained with fluorescent dyes, and the ratio of live/dead cells was analyzed by flow cytometry. (**B**) Brains were removed from mice and dissociated with 1 ml of one of five dissociation enzymes for 30 min at 37°C. Single cells were purified by density centrifugation and stained with anti-A2B5, -O4, and -O1 antibodies for 30 min on ice. Positive cellular staining was quantified by flow cytometry. (**C**) Single cell suspensions were purified from Accutase-dissociated CNS tissue by density centrifugation or bead-based myelin removal technology. Representative forward (x-axis) and side (y-axis) scatter of light plots are illustrated for each fraction following purification. (**D**) Total numbers of cells in each fraction was determined by haemocytometer. (**E–G**) Single cell fractions, following density centrifugation (**E–G**) or myelin removal bead (**F**) purification of CNS tissue, were stained with anti-A2B5, -PDGFRα, -NG2, -O4, -CC1, or O1 for 30 min on ice. Percentages of positive cellular staining were quantified by flow cytometry and absolute cell numbers were back calculated from total cell counts. Data are representative of three independent experiments

We began by optimizing technical parameters specifically for working with CNS tissues, examining total cell recovery from the mouse brain and antigen integrity following multiple tissue-dissociating enzyme and lipid debris removal techniques. All tested enzymes returned more viable cells than manual dissociation alone (**Fig. 2A**). The protease/collagenase blend Accutase yielded the highest number of viable cells per brain (∼1.6×10^6^ cells/350 mg brain). The highest retention of oligodendroglial antigens was found following Accutase or papain dissociation (**Fig. 2B**). Although papain yielded slightly more A2B5^+^ immature OPCs, Accutase dissociation produced the highest total cell yield with good overall oligodendroglial antigen preservation, thus we used Accutase for remaining experiments.

We next compared techniques for cell purification from dissociation debris using a 30/70% Percoll gradient, a linear 40% Percoll centrifugation, and commercially available magnetic beads for eliminating myelin debris. Forward and side scatter data revealed a dramatic enrichment of cells with all three techniques (**Fig. 2C**). Highest total cell yields were similarly recovered from the pellet of the 40% Percoll separation and the myelin-removed fraction following the myelin removal bead protocol (∼1.5×10^6^ cells, **Fig. 2D**). Recovery of stage-specific oligodendroglial populations was next compared by purification technique (**Fig. 2E–G**). The linear 40% Percoll purification produced the cleanest separation with the pellet containing the majority of OLCs and the fewest cells lost in the debris fraction. For the remaining experiments we used a linear 40% Percoll separation to purify single cells from the CNS.

### Optimization of oligodendroglial antibodies for flow cytometric analysis

**Figure 3:**
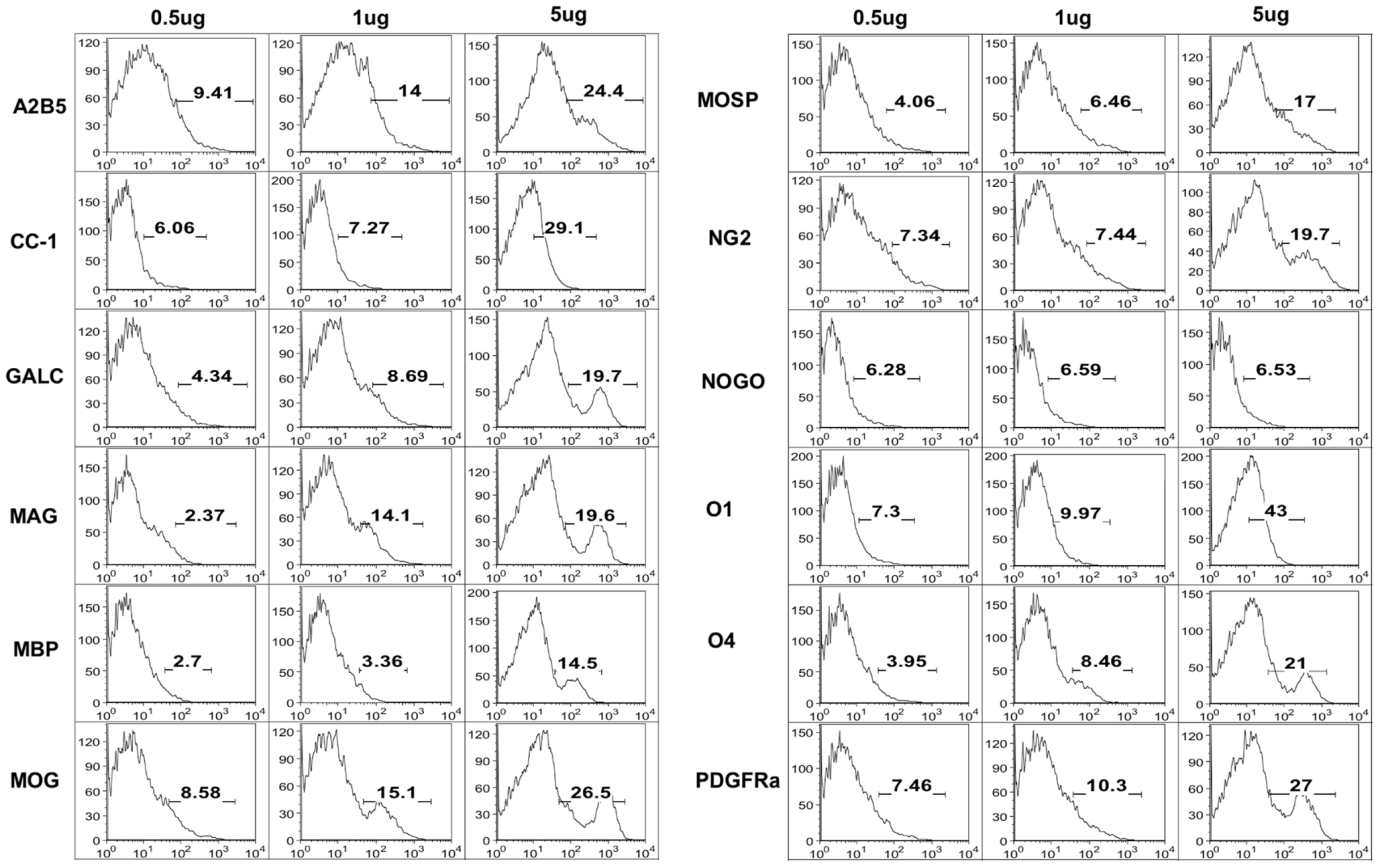
Oligodendrocyte lineage cell antibody titrations. Brains were removed from C57BL/6 mice, coarsely chopped, and dissociated with 1 ml of Accutase for 30 min at 37°C. Digested tissues were filtered, and single cells were purified using density centrifugation. Cells were stained with 0.5 µg, 1 µg, or 5 µg of each antibody or isotype control antibody for 30 min on ice. Positive cellular staining was quantified by flow cytometry.

We next sought to determine the suitability of commercially available OLC antibodies for use with the CNS preparation protocol. Twelve purified oligodendroglial antibodies were conjugated to fluorescein isothiocyanate (FITC) and titrated for flow cytometry using CNS cells prepared as described (**Fig. 3**). Antibodies and the optimal concentrations that demonstrated clear positive staining and/or selectively defined a specific stage in oligodendrocyte development were chosen for validation experiments and application in mouse models of demyelinating disease (**Table 1**). Primary antibodies were assigned to specific fluorochromes for conjugation to allow for assembly of antibody staining cocktails for surveying oligodendroglial cells throughout the lineage in a single sample.

### Validation of oligodendroglial flow cytometry using in vitro cultures and sorted cells

**Figure 4:**
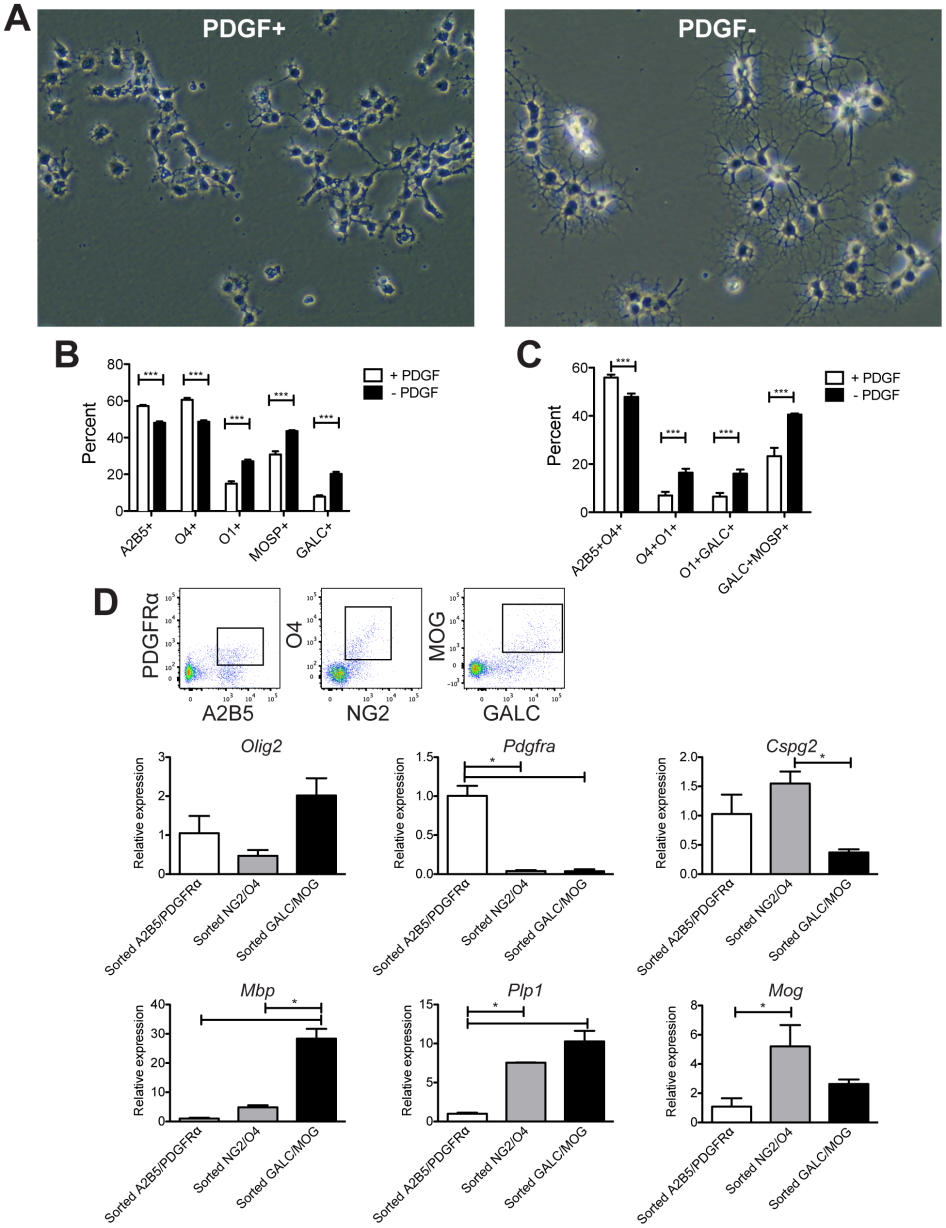
Oligodendrocyte lineage analysis by flow cytometry using cultured oligodendrocyte progenitor cells (OPCs) and differentiated oligodendrocytes. Primary OPCs were isolated from C57BL/6 postnatal day five pups by O4 immunopanning and grown for six days in the presence of or five days in the absence of the mitogen PDGF. (A) Representative photomicrographs of cultures. (B) Cultured OPCs and oligodendrocytes were lifted from plates, stained with antibodies against A2B5, O4, O1, MOSP, and GALC, and analyzed by flow cytometry. (C) Double labeling analysis. Gates were drawn for double positive populations: A2B5^+^O4^+^ intermediate OPCs, O4^+^O1^+^ late OPCs, O1^+^GALC^+^ and GALC^+^MOSP^+^ mature oligodendrocytes. (D) Adult C57BL/6 CNS cells were isolated, stained with oligodendroglial antibodies, and sorted by FACS into three populations: A2B5^+^PDGFRα^+^ early OPCs, NG2^+^O4^+^ intermediate OPCs, GALC^+^MOG^+^ mature oligodendrocytes. Representative sorting flow plots are shown. mRNA was purified and transcription levels of stage-specific oligodendroglial genes *Olig2, Pdgfra, Cspg4, Plp1, Mbp*, and *Mog* were quantified by RT-PCR. Data were normalized to a housekeeping gene and are reported relative to the A2B5^+^PDGFRα^+^ sorted population. Error bars indicate SD. *P≤0.05; **P≤0.01; ***P≤0.001 for post-hoc analysis. Data are representative of three independent experiments.

We first validated OLC-specific antibodies using highly purified cultures of OPCs and differentiated oligodendrocytes. Primary OPCs were isolated from postnatal mouse brains, selected for by O4^+^ immunopanning, and cultured in the presence of the recombinant OPC mitogen platelet-derived growth factor (PDGF). Oligodendrocyte differentiation was induced in some cultures by withdrawal of PDGF. Cells grown in the presence of PDGF demonstrated a characteristic morphology of short, bipolar processes, whereas cells grown without PDGF exhibited multiple, elaborate processes characteristic of differentiating oligodendrocytes (**Fig. 4A**). Flow cytometric analysis using antibodies spanning the oligodendrocyte lineage revealed significantly higher expression of OPC markers A2B5 and O4 in the OPC cultures (**Fig. 4B**). Differentiating oligodendrocytes expressed significantly higher expression of late OPC and mature oligodendrocyte markers O1, MOSP, and GALC. Analyzing co-expression revealed significantly more A2B5^+^O4^+^ early OPCs in the cultures with PDGF and significantly more O4^+^O1^+^ late OPCs and O1^+^GALC^+^/GALC^+^MOSP^+^ mature oligodendrocytes in the cultures without PDGF (**Fig. 4C**).

To further test the efficacy of the flow cytometric method for characterizing cell populations, we used a similar panel of OLC-specific antibodies to FACS sort oligodendroglial populations from adult mouse brains (**Fig. 4D**). RT-PCR analysis revealed all sorted OLC populations expressed the oligodendrocyte lineage marker *Olig2*. A2B5^+^PDGFRα^+^ early OPCs expressed significantly more *Pdgfra* (∼25-fold) than NG2^+^O4^+^ intermediate OPCs and GALC^+^MOG^+^ mature oligodendrocytes reflecting the down-regulation of PDGFRα during maturation. *Cspg4*, the transcript for the NG2 glycoprotein, was expressed at highest levels in intermediate OPCs, significantly greater than in mature oligodendrocytes (∼3-fold). Transcripts for myelinating oligodendrocyte markers *Mbp* and *Plp1* were expressed at highest levels in the mature oligodendrocytes. *Mbp*, *Plp1*, and *Mog* were all significantly greater in late OPCs or mature oligodendrocytes compared to early OPCs. The genes *Rbfox3* and *Gfap*, encoding for neuron-specific nuclear protein NeuN and astrocyte-specific GFAP respectively, were undetectable in all sorted populations.

### Oligodendroglial characterization by flow cytometry during postnatal development

**Figure 5:**
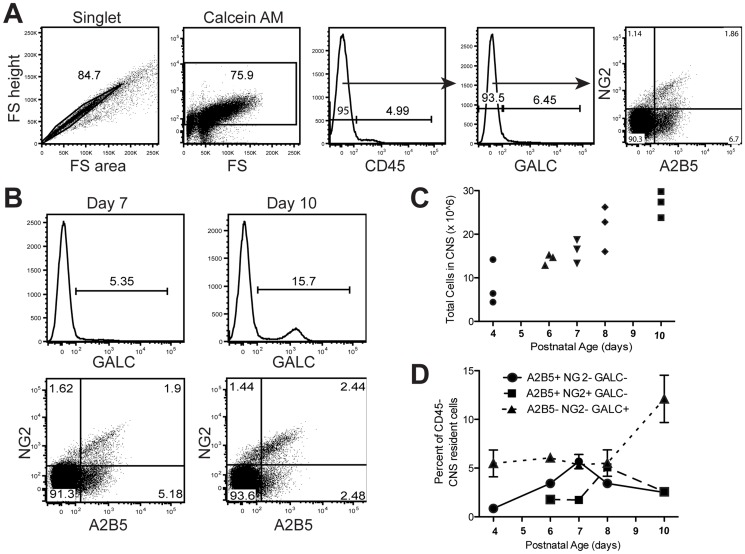
Oligodendroglial lineage analysis during development. Postnatal day four through ten C57BL/6 mice were sacrificed, brains extracted, and CNS tissue prepared for flow cytometry (n≥3/time point). Cells were stained with an antibody panel consisting of anti-A2B5, -NG2, -GALC, and -CD45 and analyzed by flow cytometry to characterize changes in oligodendroglial populations. (**A**) To examine CNS resident cells, single cells were gated, followed by live cells, followed by CD45^−^ gate. The GALC^+^ mature oligodendrocyte population was defined, and the A2B5^+^ and NG2^+^ oligodendrocyte progenitor cell (OPC) populations were defined from the GALC^−^ gate. (**b**) Representative oligodendroglial analysis from postnatal day 7 and 10 mice. Upper histograms illustrate GALC^+^ mature oligodendrocyte staining. Lower plots indicate A2B5^+^NG2^−^ early OPC and A2B5^+^NG2^+^ intermediate OPC populations. (**C**) Total cell yields from brains of individual mice, day 4 vs. day 10, *P* = 0.0013. (**D**) A2B5^+^NG2^−^GALC^−^ early OPCs (circles), A2B5^+^NG2^+^GALC^−^ intermediate OPCs (squares), and A2B5^−^NG2^−^GALC^+^ mature oligodendrocytes (triangles) are depicted. Error bars represent SD of mice per time point. Early OPCs: day 4 vs. 7, *P*≤0.001; day 7 vs. 10, *P*≤0.01. Intermediate OPCs: day 7 vs. 8, *P*≤0.001; day 8 vs. 10, *P*≤0.001. Mature oligodendrocytes: day 8 vs. 10, *P*≤0.05. Data are representative of three independent experiments.

Developmental oligodendrogenesis in the mouse has been well documented with OPC proliferation in the forebrain occurring early after birth followed by maturation and functional myelination rapidly increasing around postnatal day nine [14]. We employed a panel of oligodendroglial antibodies to characterize OLC populations during postnatal development and further validate the flow cytometry assay. Sequential gates were drawn to distinguish singlet cells from clumped cells then live cells from debris (**Fig. 5A**). A CD45 exclusion gate was drawn to eliminate residual hematopoietic cells and microglia. The GALC^+^ mature oligodendrocyte population was defined and the GALC^−^ population further analyzed for A2B5^+^ early OPC and NG2^+^ intermediate OPC populations. The total cells isolated from the CNS increased ∼3-fold between 4 and 10 days of age (**Fig. 5C**). A2B5^+^NG2^−^GALC^−^ early OPCs increased from postnatal day 4 to 7, and decreased from day 7 to 10 (**Fig. 5B and D**). This expansion of early OPCs at day 7 was followed one day later (day 8) by a peak in A2B5^+^NG2^+^GALC^−^ intermediate OPCs suggesting the beginning of maturation of the early OPCs. Intermediate OPCs decreased from day 8 to 10 coincident with a 3-fold increase in A2B5^−^NG2^−^GALC^+^ mature oligodendrocytes indicating further differentiation of the OPCs to mature oligodendrocytes (**Fig. 5B and D**). These changes observed by flow cytometry correlated with previous fate-mapping studies [14].

### Oligodendroglial characterization by flow cytometry in cuprizone-induced demyelination

We next applied the flow cytometry assay for rapid analysis of oligodendroglial populations in a well-characterized animal model of demyelinating disease. The cuprizone model, induced by feeding mice the neurotoxic copper chelator cuprizone, produces robust demyelination with substantial loss of oligodendrocyte cell bodies in the brain over the course of five weeks followed by rapid remyelination following cuprizone removal [6]. The infiltration of peripheral immune cells, and thus skewing of resident cell quantification, is largely absent making this model ideal to test flow cytometric characterization of oligodendroglial populations.

**Figure 6:**
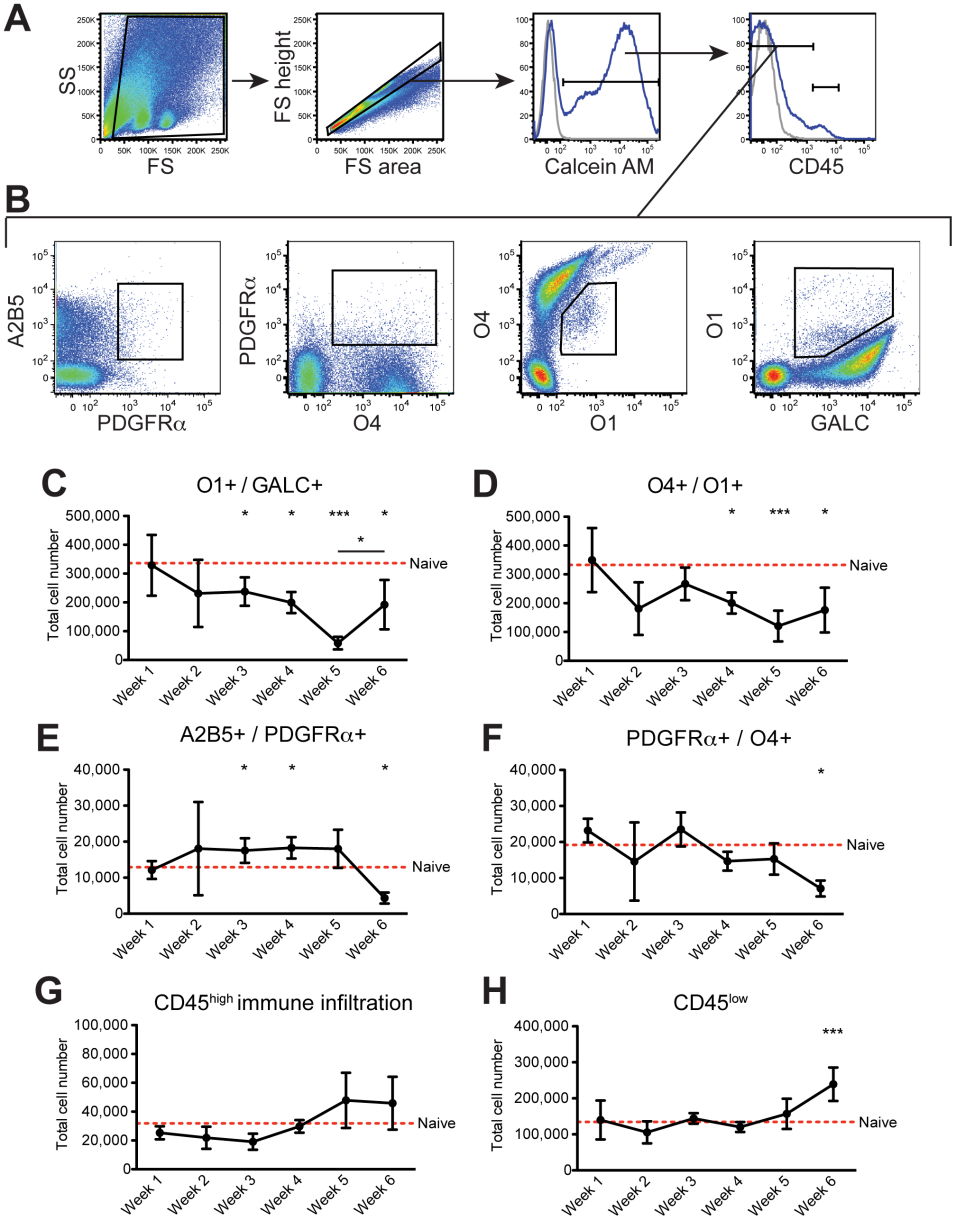
Characterizing oligodendroglial population changes through cuprizone-induced demyelination. C57BL/6 mice were fed 0.2% cuprizone chow for five weeks and returned to a normal diet for a sixth week. A cohort of mice was sacrificed weekly throughout the disease course, brains were extracted, and CNS processed for flow cytometry (n = 5). Naïve mice were concurrently assayed at each time point for standardization (n = 3). (**A**) Cells were first gated by forward and side scatter then single cells were gated. Live cells were next gated, and hematopoietic cells excluded by gating on CD45^-/low^ cells. (**B**) From the CNS resident cells, oligodendroglial populations were defined: A2B5^+^PDGFRα^+^ early OPCs, PDGFRα^+^O4^+^ intermediate OPCs, O4^+^O1^+^ pre-myelinating oligodendrocytes, and O1^+^GALC^+^ mature oligodendrocytes. Mature oligodendrocytes (**C**), pre-myelinating oligodendrocytes (**D**), early OPCs (**E**), and late OPCs (**F**) were compared to naïve mice at each time point. CD45^high^ peripheral immune cells (**G**) and CD45^low^ microglia (**H**) were also quantified. Error bars indicate SD of mice per time point. *P≤0.05; **P≤0.01; ***P≤0.001 for post-hoc analysis. Data are representative of two independent experiments.

C57BL/6 mice were fed cuprizone for five weeks and returned to normal chow for the sixth week. A cohort of mice was sacrificed weekly throughout the disease course and following remyelination. To analyze additional oligodendroglial subsets and demonstrate the versatility of the staining protocol in incorporating different markers, the panel was expanded and modified to include five antigens across the lineage. Sequential gates were drawn to first distinguish intact cells from cellular debris based on size and granularity (**Fig. 6A**). Next singlet cells were gated followed by live cells. Finally the CD45^-/low^ fraction was analyzed to exclude hematopoietic cells. From the CNS-resident fraction, oligodendroglial populations were defined by double positive staining to exclude significant overlap between populations (**Fig. 6B**). Naïve age-matched mice were likewise assayed at each time point for standardization of population gating. The loss of O1^+^GALC^+^ mature oligodendrocytes was quite variable among individual mice in both experiments performed at week 2, but by the third week of cuprizone, there were significantly fewer O1^+^GALC^+^ mature oligodendrocytes in the CNS than in naïve controls (**Fig. 6C**). The loss of mature oligodendrocytes was maintained at four and five weeks. There was likewise a significant loss of O4^+^O1^+^ pre-myelinating oligodendrocytes at four and five weeks of cuprizone (**Fig. 6D**). Surveying the progenitor populations, A2B5^+^PDGFRα^+^ early OPCs were significantly expanded after three and four weeks (**Fig. 6E**). The number of PDGFRα^+^O4^+^ intermediate OPCs, however, never varied from naïve controls throughout the demyelinating time course suggesting lack of differentiation of the early OPCs (**Fig. 6F**). Following cuprizone withdrawal, the number of early and late OPCs in the CNS fell dramatically to fewer than in naïve mice. Concurrently the number of mature oligodendrocytes increased significantly suggesting a maturation of the expanded OPC populations. Despite partial recovery the number of mature and pre-myelinating oligodendrocytes did not reach naïve levels following one week of remyelination.

Examining the CD45^+^ population, we confirmed no significant infiltration of peripheral CD45^high^ immune cells and no change in the number of CD45^low^ microglia during the course of demyelination (**Fig. 6G and H**). This was unexpected given previous reports of increased microglia in the corpus callosum following cuprizone and may reflect a sub-optimal isolation procedure for microglial analysis [15]. Interestingly, recovered CD45^low^ microglia increased during the remyelinating phase suggesting a correlation with remyelination as supported by other myelin repair models [16].

### Oligodendroglial characterization in EAE demyelinating disease

In SJL/J mice EAE induced by priming with the PLP_139–151_ peptide results in a relapsing-remitting clinical disease associated with substantial demyelination throughout the CNS. Similar to MS, EAE bears a strong inflammatory component characterized by mass pathogenic immune cell infiltration into the spinal cord and corresponding motor deficits [17]. To prevent masking of OLC changes by subtler changes in the brain, we specifically analyzed OLCs in the spinal cord. Furthermore to prevent skewing due to immune cell infiltration, a hematopoietic marker and an exclusion gate were incorporated to specifically characterize CNS resident cells.

**Figure 7:**
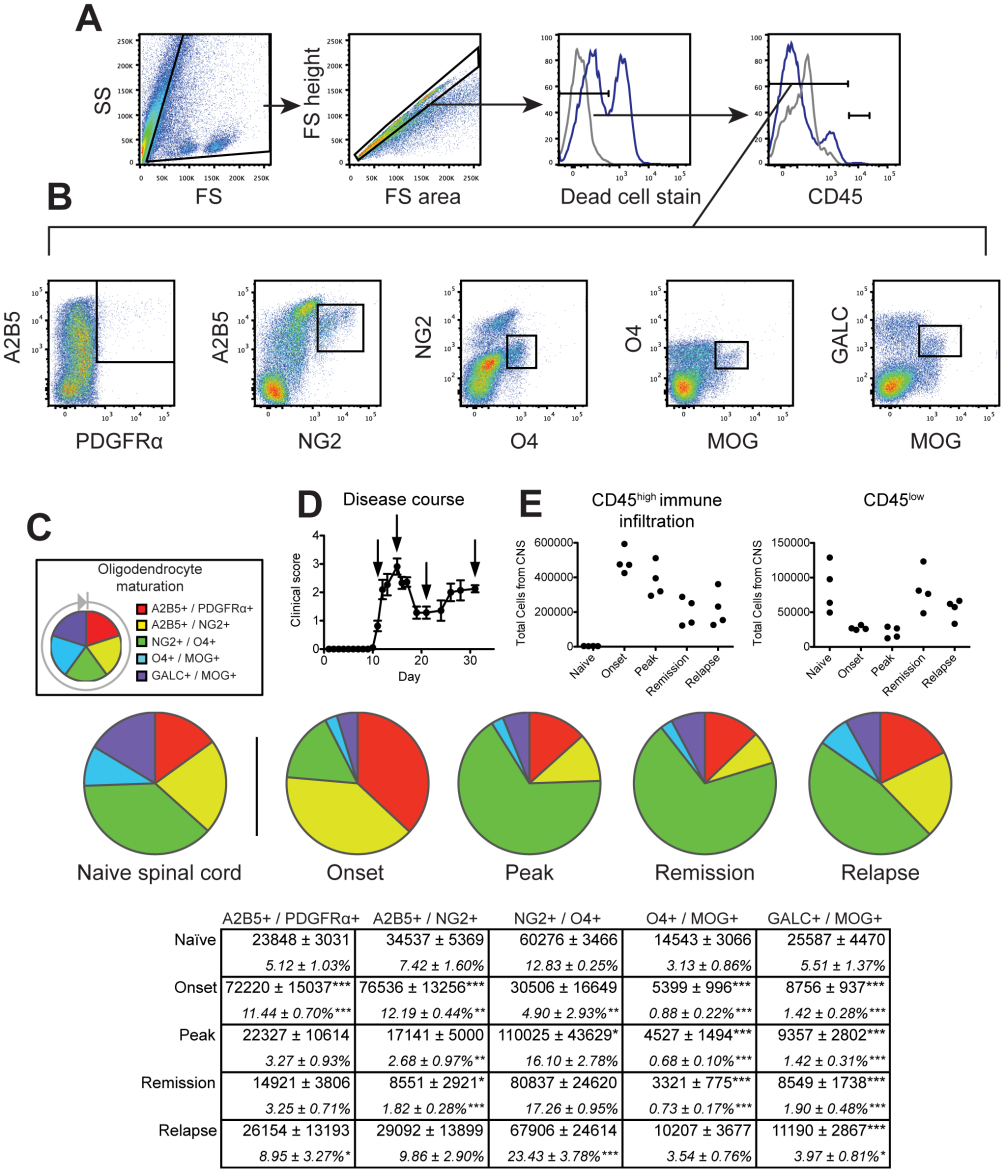
Characterizing oligodendroglial populations through relapsing-remitting EAE. SJL/J mice were immunized with PLP_139–151_ and scored daily for clinical disease. A cohort of mice was sacrificed at disease onset, peak, remission, and relapse, and spinal cords were analyzed by flow cytometry (n = 5). Naïve mice were also assayed at each time point for standardization across days (n = 3). (**A**) Cells were distinguished from debris by forward and side scatter then singlet cells were gated. Live cells were gated by dead cell exclusion, and CNS resident cells were identified as CD45^−^ or CD45^low^. (**B**) Oligodendroglial cells were defined by double positive staining: A2B5^+^PDGFRα^+^ early OPCs, A2B5^+^NG2^+^ intermediate OPCs, NG2^+^O4^+^ late OPCs, O4^+^MOG^+^ pre-myelinating oligodendrocytes, and GALC^+^MOG^+^ mature oligodendrocytes. (**C**) The complete oligodendroglial lineage is illustrated for the naïve spinal cord. Oligodendroglial populations at each stage of disease were compared to naïve spinal cord. The table indicates number and percentage of CD45^-/low^ CNS resident cells ± SD per group of mice. (**D**) Clinical relapsing-remitting disease course and sacrifice points. (**E,F**) CD45^high^ infiltrating immune cells and CD45^low^ microglia in the spinal cord were also quantified throughout EAE disease. *P≤0.05; **P≤0.01; ***P≤0.001 for post-hoc analysis. Data are representative of three independent experiments.

Relapsing-remitting EAE was induced in SJL/J mice by priming with the PLP_139–151_ peptide, and mice were scored daily for clinical disease. At specific disease time points: onset, peak, remission, and relapse, a cohort of mice was sacrificed for OLC analysis in the spinal cord by flow cytometry (**Fig. 7D**). Naïve age-matched control mice were also assayed at every time point for standardization of population gating. A similar gating scheme as that used in cuprizone experiments was employed to exclude debris, clumped cells, dead cells, and, importantly, CD45^high^ infiltrating cells from OLC analysis (**Fig. 7A**). We further expanded the staining panel to incorporate labeling a sixth antigen additionally demonstrating the flexibility of the method in investigating specific populations (**Fig. 7B**). Surveying the entire lineage in the CNS-resident cells recovered from the naïve spinal cord we found the isolate comprised of roughly 25% mature and pre-myelinating oligodendrocytes and 75% OPCs (**Fig. 7C**).

The naïve mouse spinal cord contained very few CD45^high^ infiltrating cells whereas EAE disease from onset through relapse was associated with robust immune cell infiltration (∼30–50% of total recovered cells, **Fig. 7E**). The CD45^low^ population decreased at onset and peak of disease consistent with previous studies demonstrating activated microglia upregulate CD45 during EAE (**Fig. 7F**) [18]. At onset of clinical EAE, there was a significant loss of GALC^+^MOG^+^ mature and O4^+^MOG^+^ pre-myelinating oligodendrocytes coincident with a dramatic expansion of A2B5^+^PDGFRα^+^ early and A2B5^+^NG2^+^ intermediate OPCs (**Fig. 7C**). This loss of mature oligodendrocytes never fully recovered through the rest of the disease course. As mice reached the peak of clinical disease, the NG2^+^O4^+^ late OPC population was significantly increased suggesting a partial maturation of the previously expanded early/intermediate OPC pool (**Fig. 7C**). This expanded late OPC population was maintained through remission and relapse suggesting an arrest in differentiation to mature oligodendrocytes. At disease relapse early OPCs increased to similar levels as in the naïve spinal cord suggesting a correlation between early OPC expansion and inflammatory status in the CNS or alternatively a delayed return of OPCs to naïve levels following disease remission.

## Discussion

Despite the routine use of flow cytometry for circulating hematopoietic cell analysis and immune infiltration of the CNS, the assay remains largely unexplored for investigation of resident CNS cells. In two 1988 studies, Dubois-Dalcq and colleagues first used FACS to sort OPCs from rat cerebrum for culture using an antibody against A2B5 [19], [20]. Since then a handful of studies have used FACS of rodent CNS tissue to sort various single populations for culture or downstream investigation [21]–[25]. Other groups have used flow cytometry to quantify single CNS populations *in vivo* including A2B5^+^ OPCs or MOG^+^, MBP^+^, or CNPase^+^ oligodendrocytes [26]–[28]. However, this is the first study optimizing and fully characterizing cells of the oligodendroglial lineage in mouse CNS by flow cytometry using multiple OLC markers.

The trepidation in utilizing flow cytometry likely stems from four major obstacles encountered when working with CNS tissue: complex cytoarchitecture, high lipid content, oligodendroglial antigen preservation, and oligodendroglial antibody specificity and format. In this work we have optimized a protocol to overcome each of these barriers allowing for effective analysis of OLCs.

The CNS is a largely immobile tissue with a highly complex cytoarchitecture. Successful flow cytometry requires samples to be focused in a stream of single cells and thus solid organs must be dissociated into individual intact cells. We found Accutase most effectively dissociated viable cells from the mouse CNS. The CNS is also a lipid-rich tissue precisely because of the abundant myelin that oligodendrocytes produce. Fatty tissues pose a major barrier to flow cytometry as lipid debris can clog the apparatus. Additionally myelin debris is highly auto-fluorescent and can confound meaningful spectral data. We found a linear 40% Percoll spin efficiently purified cells with minimal loss in the debris fraction. Dissociation enzymes cleave proteins ubiquitously, thus it was necessary to identify an enzyme that preserved oligodendroglial antigen integrity. It was also necessary to identify a purification technique that spared OLC antigens located on the cell bodies. Unlike other enzymes that either degraded OLC antigens or affected viability of specific populations, we found that Accutase dissociation allowed for recovery of a high number of viable cells and most efficiently preserved oligodendroglial antigens. A linear 40% Percoll spin most effectively isolated both OPC and oligodendrocyte cell bodies expressing the oligodendroglial antigens of interest.

Oligodendroglial antibodies for flow cytometry should ideally meet several requirements: 1) bind antigens restricted to OLCs specifically in a narrowly defined stage in oligodendrocyte development, 2) bind epitopes on the extracellular surface of the cell body, and 3) be monoclonal antibodies of different classes and/or host species. Through titration experiments we were able to identify a number of oligodendroglial antibodies commonly used for IHC and ICC that labeled a population detectable by flow cytometry. Many of these antibodies bound extracellular epitopes preventing the need for cellular fixation and permeabilization that can degrade antigens. To restrict labeling to CNS-resident cells, we incorporated anti-CD45 antibody to exclude hematopoietic cells from analysis. To further definitively describe stage-specific OLCs and exclude prospective binding to non-oligodendroglial cells, we defined all populations of interest by double labeling. We were able to confirm the success of this strategy in identifying precise windows in OLC development both *in vitro* using defined OPC and oligodendrocyte cultures, and *in vivo* through RNA analysis of the populations and OLC characterization during developmental myelination. Many of the key antibodies were monoclonal IgM's raised in mouse preventing the use of anti-Ig class or anti-species secondary antibodies in a panel. We thus directly conjugated each antibody to a unique fluorochrome enabling the detection of eight different proteins expressed throughout the lineage in a single CNS. The ease of the antibody conjugation step allows for panels to be easily modified to incorporate different oligodendroglial or other cell markers, and the variety of available conjugating fluorochromes allows for tailoring to specific flow cytometers with different spectral configurations.

The value of this method lies in its speed and unbiased analysis. The assay can be reasonably performed, from live mouse to data collection, on upwards of 15 mice in a single day. All gating for population analyses is defined by control samples and directly applied to experimental samples in an impartial fashion. A single researcher can objectively characterize OLC populations in individual brains and make comparisons between groups of mice or brain regions in a few days. Additionally antibody cross-reactivity with antigens in humans has enabled us to preliminarily characterize oligodendroglial populations in human tissue samples. Most importantly we demonstrated the broad utility of the method during developmental myelination and in two distinct models of demyelination. Demyelination following cuprizone intoxication has been well documented, and our results corresponded well with previous studies indicating the sensitivity of this assay [6]. Interestingly in EAE myelination levels did not correspond with clinical disease severity highlighting the crucial need for sensitive assays like the flow cytometry method to assess demyelination *per se*. In addition to demyelination we observed changes in OPC populations in both disease models suggestive of remyelination or attempts thereof. The increase in early OPCs during cuprizone treatment was followed by a dramatic reduction and concomitant increase in mature oligodendrocytes following cuprizone withdrawal suggesting active maturation of the OPCs. During EAE we detected a substantial increase in OPCs coinciding with disease onset that was followed by an expansion of the late OPC population at disease peak suggesting a partial maturation of the previously expanded OPC pool and an attempt of the host to repair myelin. The expanded late OPC population was maintained through remission and relapse suggesting an arrest in differentiation to mature oligodendrocytes in the face of the chronic autoimmune T cell-mediated inflammatory response and an unsuccessful attempt at remyelination. The early OPC population was significantly increased again from disease remission to relapse suggesting a correlation between early OPC expansion and clinical disease symptoms in EAE, or, alternatively could reflect a delayed return to baseline following reduced inflammation and amelioration of disease during the remission.

In addition to OLC data, the CD45^+^ staining provided insight into the immune compartment, most notably reflecting a dramatic infiltration of CD45^high^ immune cells in EAE but not the cuprizone model. Additionally microglial activation and up-regulation of CD45 in acute EAE is consistent with our observed decrease in CD45^low^ and concomitant increase in CD45^high^ numbers. Future optimization of microglial analysis using flow cytometry, e.g. quantifying M1 vs. M2 microglia, as well as other resident CNS and immune cell types will greatly expand the utility of the method for use in numerous other animal models.

Flow cytometry has proven to be a powerful tool for immunologists for decades. Here we demonstrated flow cytometry to be an effective, sensitive, and reliable means to rapidly enumerate oligodendroglial cells during disease, thus functionally providing a rapid global analysis of myelin-producing cells in adult mouse brain and providing important information regarding effects of disease on OPC proliferation/differentiation which is useful for defining the pathogenesis and therapy of MS.

## Supporting Information

Table S1 Enzymes tested for CNS dissociation.
(DOCX)

Table S2 Primers used for real-time RT-PCR. Single cells isolated and purified from adult mouse CNS were sorted by FACS: A2B5^+^PDGFRα^+^ early OPCs, NG2^+^O4^+^ intermediate OPCs, GALC^+^MOG^+^ mature oligodendrocytes. mRNA was purified and transcription levels of the stage-specific oligodendroglial genes listed were quantified by RT-PCR.
(DOCX)
