# Supplementary material for: Characterization of Oligodendroglial Populations in Mouse Demyelinating Disease Using Flow Cytometry: Clues for MS Pathogenesis
Source: PLoS One. 2014 Sep 23;9(9):e107649. doi: 10.1371/journal.pone.0107649 (PMC4172589; doi:10.1371/journal.pone.0107649)
Supplement: Table S1 — Enzymes tested for CNS dissociation. (DOCX) [file pone.0107649.s001.docx]

| Enzyme | Concentration | Manufacturer |
| --- | --- | --- |
| Accutase | neat | EMD Millipore |
| Liberase DL | 100 μm/ml | Roche |
| Liberase DH | 100 μm/ml | Roche |
| Liberase TL | 100 μm/ml | Roche |
| Liberase TM | 100 μm/ml | Roche |
| Liberase TH | 100 μm/ml | Roche |
| Papain | 200 U | Worthington Biochemical |
| TrypLE | neat | Life Technologies |
| Trypsin-EDTA | 0.25% | Life Technologies |
