# Supplementary material for: Characterization of Oligodendroglial Populations in Mouse Demyelinating Disease Using Flow Cytometry: Clues for MS Pathogenesis
Source: PLoS One. 2014 Sep 23;9(9):e107649. doi: 10.1371/journal.pone.0107649 (PMC4172589; doi:10.1371/journal.pone.0107649)
Supplement: Table S2 — Primers used for real-time RT-PCR. Single cells isolated and purified from adult mouse CNS were sorted by FACS: A2B5+PDGFRα+ early OPCs, NG2+O4+ intermediate OPCs, GALC+MOG+ mature oligodendrocytes. mRNA was purified and transcription levels of the stage-specific oligodendroglial genes listed were quantified by RT-PCR. (DOCX) [file pone.0107649.s002.docx]

| Common protein name | Gene symbol | RefSeq Accession no. | Amplicon size (bp) | Amplicon position in the RefSeq sequence |
| --- | --- | --- | --- | --- |
| Neuron-glial antigen 2 (NG2) | *Cspg4* | NM_139001.2 | 179 | 1726 |
| Glial fibrillary acidic protein | *Gfap* | NM_010277.3 | 150 | 495 |
| Myelin basic protein | *Mbp* | NM_010777.3 | 129 | 732 |
| Myelin oligodendrocyte glycoprotein | *Mog* | NM_010814.2 | 71 | 883 |
| Oligodendrocyte transcription factor 2 | *Olig2* | NM_016967.2 | 88 | 940 |
| Platelet-derived growth factor receptor, alpha | *Pdgfra* | NM_011058.2 | 65 | 3404 |
| Proteolipid protein 1 | *Plp1* | NM_011123.2 | 119 | 431 |
| Neuronal nuclei (NeuN) | *Rbfox3* | NM_001024931.2 | 123 | 645 |
|  |  |  |  |  |
| **Housekeeping genes** |  |  |  |  |
| Phosphoglycerate kinase 1 | *Pgk1* | NM_008828.2 | 108 | 740 |
| Ribosomal protein L13a | *Rpl13a* | NM_009438.5 | 135 | 721 |
